# FireProt: Energy- and Evolution-Based Computational Design of Thermostable Multiple-Point Mutants

**DOI:** 10.1371/journal.pcbi.1004556

**Published:** 2015-11-03

**Authors:** David Bednar, Koen Beerens, Eva Sebestova, Jaroslav Bendl, Sagar Khare, Radka Chaloupkova, Zbynek Prokop, Jan Brezovsky, David Baker, Jiri Damborsky

**Affiliations:** 1 Loschmidt Laboratories, Department of Experimental Biology and Research Centre for Toxic Compounds in the Environment RECETOX, Masaryk University, Brno, Czech Republic; 2 International Clinical Research Center, St. Anne's University Hospital Brno, Brno, Czech Republic; 3 Department of Information Systems, Faculty of Information Technology, Brno University of Technology, Brno, Czech Republic; 4 Department of Chemistry and Chemical Biology, Rutgers University, Piscataway, New Jersey, United States of America; 5 Enantis, Ltd., Brno, Czech Republic; 6 Department of Biochemistry, University of Washington, Seattle, Washington, United States of America; University of North Carolina at Chapel Hill, UNITED STATES

## Abstract

There is great interest in increasing proteins’ stability to enhance their utility as biocatalysts, therapeutics, diagnostics and nanomaterials. Directed evolution is a powerful, but experimentally strenuous approach. Computational methods offer attractive alternatives. However, due to the limited reliability of predictions and potentially antagonistic effects of substitutions, only single-point mutations are usually predicted *in silico*, experimentally verified and then recombined in multiple-point mutants. Thus, substantial screening is still required. Here we present FireProt, a robust computational strategy for predicting highly stable multiple-point mutants that combines energy- and evolution-based approaches with smart filtering to identify additive stabilizing mutations. FireProt’s reliability and applicability was demonstrated by validating its predictions against 656 mutations from the ProTherm database. We demonstrate that thermostability of the model enzymes haloalkane dehalogenase DhaA and γ-hexachlorocyclohexane dehydrochlorinase LinA can be substantially increased (Δ*T*_m_ = 24°C and 21°C) by constructing and characterizing only a handful of multiple-point mutants. FireProt can be applied to any protein for which a tertiary structure and homologous sequences are available, and will facilitate the rapid development of robust proteins for biomedical and biotechnological applications.

## Author Summary

Proteins are increasingly used in numerous biotechnological applications. A key property determining proteins’ applicability is their stability under operating conditions. Natural proteins can be stabilized by modification of their structure. Methods of molecular biology allow introduction of modifications–mutations–to the protein structure at will, but it is not straightforward where to mutate and which amino acid to introduce for better stability. Computational methods can be used for prediction of stabilizing mutations using computers. Current computational methods predict libraries of single-point mutations, which need to be constructed individually, tested and recombined, resulting in non-trivial experimental effort. Here we present a robust computational strategy for predicting multiple-point mutants, providing extremely stabilized proteins with a minimal experimental effort.

## Introduction

Proteins are increasingly used in biotechnological applications as therapeutics [1], diagnostics [2], nanomaterials [1] and biocatalysts [3]. Despite numerous advantages, the utility of proteins is frequently restricted by their limited stability under practical conditions, such as high temperatures, extreme pH, or the presence of organic solvents or proteases. Their thermostability is usually positively correlated with stability and performance in the presence of denaturing agents [4], expression yield [5], serum survival time [6] and shelf-life [7]. Thus, it is a key determinant of proteins’ applicability in biotechnological processes. High temperatures may also be required to prevent bacterial contamination during enzymatic food processing [8]. Moreover, thermostable proteins can tolerate much larger numbers of mutations than mesophilic variants and show enhanced evolvability in protein engineering projects [9].

Protein engineering is frequently applied to obtain more stable proteins. If successful, such efforts typically enhance the melting temperature (*T*_m_) of engineered proteins by 2 to 15°C [7, 10]. Extremely stabilized proteins with even greater increases in melting temperature (Δ*T*_m_) have been engineered by incorporating multiple mutations, and several outstanding increases of up to 35°C have been achieved using directed evolution methods [8]. However, these methods generally require extensive experiments, including screening up to 10^8^ colonies of organisms expressing mutant variants to identify stable constructs, and appropriate high-throughput screening assays must be available [11]. A currently popular strategy is saturated mutagenesis of hotspots identified by (semi-)rational approaches [7, 8, 12], such as the most flexible residues [10]; tunnel-forming residues [13]; or residues at multimeric interfaces [14]. The selected hotspots are then subjected to site-saturation mutagenesis (while leaving the rest of the protein unchanged) to create smaller smart libraries, markedly reducing the required screening to thousands of colonies.

A long-sought alternative to screening-based approaches is reliable *in silico* design of stability-enhancing mutations. Numerous stable proteins have been computationally engineered via diverse approaches (singly or in combination), e.g., identification of back-to-consensus or ancestral mutations, calculation of changes in folding free energies upon mutation, introduction of disulfide bridges and elimination of highly flexible regions [7, 8, 12]. However, mutants generated using computational methods have rarely surpassed the 15°C Δ*T*_*m*_ threshold of outstanding stabilization as a result of neutral, destabilizing or function-corrupting mutations that were predicted as stabilizing due to moderate accuracy of these methods [15, 16]. To overcome this obstacle and provide substantial stabilization, predicted mutations are usually introduced by site-directed mutagenesis and tested individually. The most viable mutations are then recombined in multiple-point mutants assuming they have additive effects, but this is often invalid due to antagonistic epistatic effects of individual mutations [17]. For all those reasons, no computational method capable of directly designing highly stable multiple-point mutants has been previously published.

Here we introduce a strategy, FireProt, for computationally designing multiple-point mutants, enabling significant improvements of protein stability with minimal experimental effort. We demonstrate its power by stabilizing the model proteins haloalkane dehalogenase (HLD) DhaA and γ-hexachlorocyclohexane dehydrochlorinase LinA. The method’s general applicability was further verified by validation against information from the ProTherm database [18], demonstrating that it can be used to identify stabilizing mutations in diverse proteins with known tertiary structures and homologous sequences allowing phylogenetic analysis, and thus should have broad utility in protein stabilization projects.

## Results

### Development of FireProt strategy for design of stabilizing multiple-point mutants

**Fig 1:**
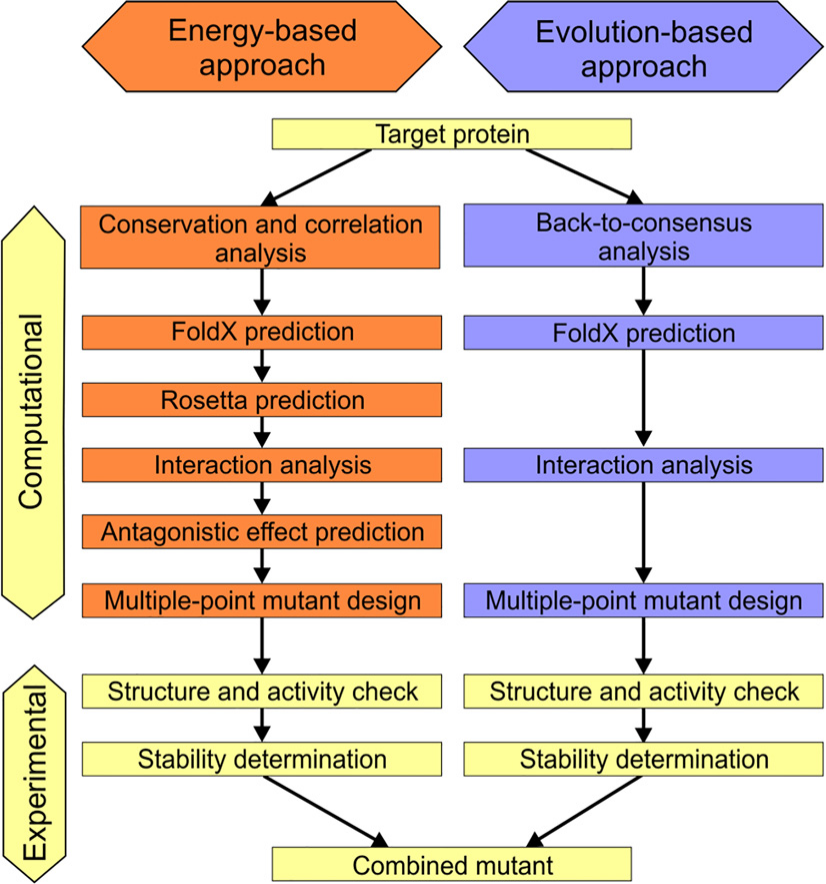
Workflow of the FireProt method. Individual steps involved in the energy- and evolution-based approaches

The FireProt strategy for protein stabilization is based on combining the best multiple-point mutants obtained from predictions of ΔΔG following mutation from a set of crystal structures and evolutionary information derived from multiple sequence alignment (Fig 1). Additional pre- and post-processing filters are applied in both approaches to improve prediction reliability and reduce the required computational effort.

#### Dataset construction

FireProt’s capacity to identify the most stabilizing single point mutations either by energy- or evolution-based approaches was evaluated using the dataset derived from the ProTherm database employing the records with absolute ΔΔG values ≥ 0.5 kcal.mol^-1^. This value was selected since the estimated experimental error for measurements of ΔΔG is about 0.48 kcal.mol^-1^ [19]. When multiple ΔΔG values were available for a single mutation, the value determined closest to the standard experimental conditions was retained. Ten most mutated proteins from the ProTherm database, representing all four protein structural classes (S1 Table), were selected to increase the chance that predicted mutations will be experimentally characterized. The total number of possible mutations in these 10 proteins was 30,058, but good quality experimental data were available for only about 2% of the mutations, yielding the dataset of 656 mutations (119 stabilizing and 537 destabilizing) at 337 different positions (S1 Table).

#### Optimization of energy-based approach

We evaluated the performance of four prediction tools: FoldX [20], Rosetta [21], ERIS [22] and CUPSAT [23], using the ProTherm dataset (S2 Table). The purpose was to select suitable combination of tools and the thresholds of predicted energy change upon mutation that would achieve very high precision (the ratio between the truly stabilizing mutations and mutations predicted as stabilizing) and simultaneously very low false positive rate (the fraction of destabilizing mutations incorrectly predicted as stabilizing from all truly destabilizing mutations). The stability change thresholds were tested in the range between -2.5 and +2.5 kcal.mol^-1^ with the step size 0.5 kcal.mol^-1^. According to this evaluation, Rosetta and FoldX achieved the highest precision, 76% and 67%, respectively, when using the thresholds of -2 kcal.mol^-1^ and -1 kcal.mol^-1^ (S2 Table). The false positive rates for these thresholds for Rosetta and FoldX were 1% and 2%, respectively (S2 Table).

#### Evaluation of energy-based approach

Combination of the prediction by the two best performing tools using their optimal thresholds with conservation analysis by Rate4Site tool [24] in the energy-based FireProt approach performed notably better than either Rosetta or FoldX alone: FireProt achieved 100% precision and 0% false positive rate. Such high reliability in prediction was achieved at the expense of the number of recognized stabilizing mutations. Rosetta and FoldX correctly identified 16 and 20 truly stabilizing mutations out of 21 and 30 mutations predicted by these tools as stabilizing (S2 Table). However, only 8 mutations were agreed upon as stabilizing by both methods following the FireProt strategy. Conservation analysis discarded 2 false positive mutations since they targeted immutable positions with high CONSURF conservation grade (≥ 8) [25, 26]. The remaining 6 mutations selected by FireProt as stabilizing were all true positives. In addition to 6 mutations included in the evaluation, the FireProt predicted other 101 stabilizing mutations for 10 most mutated proteins for which, however, experimental data were not available (S3 Table).

#### Evaluation of evolution-based approach

In addition to the energy-based approach, the evolution-based approach of FireProt strategy was also evaluated using the ProTherm dataset. The back-to-consensus method [27] identified six potentially stabilizing mutations in the 10 most mutated proteins for which experimental data were available. Four of these mutations were true positives and two were false positives. The subsequent application of the FoldX filter (Fig 1) correctly discarded one of the false positive mutations, giving a precision of 80%.

#### Evaluation of prediction for multiple-point mutants

The last step in the development of the FireProt workflow before its application towards a design of thermostable proteins was to evaluate Rosetta’s ability to predict the stability effects for multiple-point mutations. We collected a consistent dataset of previously measured stability changes in 46 mutants of DhaA enzyme for this purpose. Using this dataset a correlation of 0.81 between the predicted ΔΔG and experimentally determined *T*_*m*_ values was observed, suggesting that Rosetta could be employed for this purpose (S1 Fig).

### Design of thermostable haloalkane dehalogenase DhaA

DhaA enzyme was selected as the first model protein due to the wealth of knowledge available on mutants engineered towards higher thermostability, prolonged half-life and stability in organic co-solvents that enables quantitative comparison of their performance with the mutants designed by FireProt [13, 28].

#### Energy-based approach

**Fig 2:**
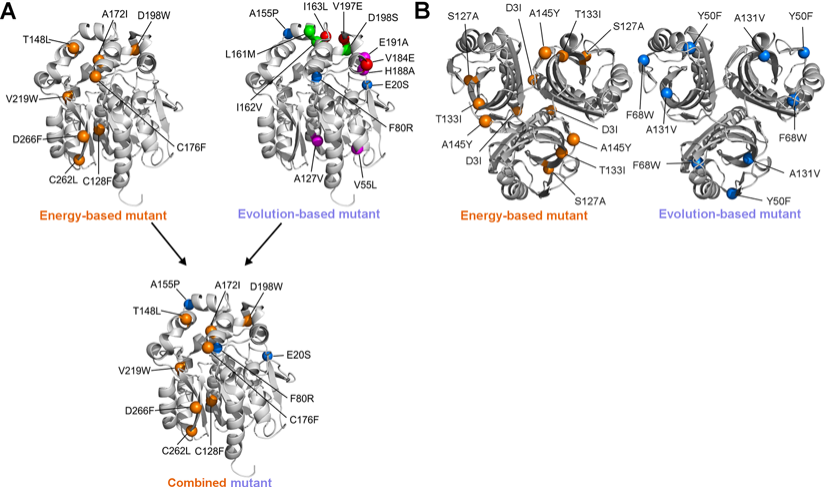
Location of stabilizing mutations in designed enzymes. A) Locations of substitutions in energy-based, evolution-based and combined mutants of DhaA enzyme. Substitutions in the multiple-point mutant designed by the energy-based approach (DhaA112) are represented as orange spheres, while substitutions in multiple-point mutants designed by the evolution-based approach are represented as red (DhaA100), blue (DhaA101), green (DhaA102) and magenta (DhaA103) spheres. Mutations in the combined mutant (DhaA115) are colored in orange and blue in correspondence with their original mutants (DhaA112 and DhaA101). B) Locations of substitutions in energy-based, and evolution-based mutants of LinA enzyme. Substitutions in the multiple-point mutant designed by the energy-based approach (LinA01) are represented as orange spheres, while substitutions in multiple-point mutant designed by the evolution-based approach (LinA02) are represented as blue spheres.

Out of 5,529 possible single-point mutations, 1,919 were at positions with high evolutionary conservation (CONSURF grade ≥ 8) or exhibiting evolutionary correlation with other residues (Correlated Mutation Analysis score ≥ 0.8 calculated by Comulator tool [29]), indicating that these positions are functionally important. All these mutations were discarded to avoid major changes in the enzymes’ activity or substrate specificity. FoldX analysis of ΔΔG for the remaining 3,610 mutations identified 151 potentially stabilizing single-point mutations. Rosetta calculation was then applied to further decrease the number of potentially false positives among these mutations, resulting in 22 promising mutations (S4 Table). A mutation disrupting a salt bridge and five with antagonistic effects (ΔΔG of double-point mutants > -3.0 kcal.mol^-1^) were also discarded, leaving 16 potentially stabilizing mutations. Only the most favorable mutations at each position were further analyzed for their mutual interactions, leaving a final set of eight potentially non-antagonistic stabilizing mutations: C128F, T148L, A172I, C176F, D198W, V219W, C262L and D266F (Fig 2). The Rosetta predicted high stability of the recombined multiple-point mutant DhaA112 carrying all these mutations (Table 1).

**Table 1:** Characteristics of predicted multiple-point mutants of DhaA.

Method	Protein	Mutations	Rosetta ΔΔG (kcal.mol^-1^)	DSC		Activity at 37°C ^b^ (nmol s^-1^ mg^-1^)
				*T*_m_ (°C)	Δ*T*_m_ (°C)	
	DhaAwt	-^a^	-^a^	49.0 ± 0.7	-^a^	18.0
Energy-based	DhaA112	C128F + T148L + A172I + C176F + D198W + V219W + C262L + D266F	-32.0 ± 1.4	65.2 ± 0.1	+16.2	5.5
Evolution-based	DhaA100	I136L + V184E + V197E	1.1 ± 0.3	48.5 ± 0.4	-0.6	9.4
	DhaA101	E20S + F80R + A155P	0.8 ± 0.1	58.6 ± 0.3	+9.6	49.3
	DhaA102	L161M + I162V + D198S	2.3 ± 0.7	48.1 ± 0.1	-0.9	9.4
	DhaA103	V55L + A127V + H188A + E191A	3.0 ± 1.0	51.4 ± 0.1	+2.3	6.5
Combined	DhaA115	E20S + F80R + C128F + T148L + A155P + A172I + C176F + D198W + V219W + C262L + D266F	-32.4 ± 1.0	73.6 ± 0.1	+24.6	5.6

^a^ not applicable

^b^ activity determined with 1-iodohexane at 37°C and pH 8.6; ΔΔG–predicted change in Free Gibbs Energy; DSC–Differential Scanning Calorimetry; DhaA115 combines mutations of DhaA101 and DhaA112

#### Evolution-based approach

The back-to-consensus approach employing simple consensus and frequency ratio predictions identified 42 potentially stabilizing substitutions (S5–S8 Tables). Of these, 22 were excluded by FoldX predictions as potentially destabilizing (ΔΔG > 0.5 kcal.mol^-1^), and seven to preserve residues with side-chains involved in important interactions. In total, 13 back-to-consensus mutations passed all of the applied filters and were combined into four multiple-point mutants (Fig 2). These mutations were combined according to their different origin from two applied consensus techniques using either representative MSA of the HLD-II subfamily or MSA of whole HLD family (see Methods for more details). DhaA100 featured the I136L, V184E and V197E mutations, which were predicted by both simple consensus and frequency ratio analyses of the representative MSA of the HLD-II subfamily. DhaA101 contained the mutations E20S, F80R and A155P, which were predicted solely by the frequency ratio analysis, while DhaA102 included the mutations L161M, I162V and D198S, which were predicted solely by the simple consensus analysis. Finally, DhaA103 contained the V55L, A127V, H188A and E191A mutations, which were predicted by simple consensus analysis of the representative MSA of the whole HLD family. Interestingly, none of these mutants was predicted to be more stable than the wild-type by Rosetta (Table 1).

#### Characterization of mutants designed by FireProt

Expression and purification of all constructed mutants resulted in good yields and protein purity. Far-UV CD spectra of wild-type and mutants show that none of the mutations caused significant changes in secondary structure (S2A Fig) and all tested variants were active with 1-iodohexane at 37°C (Table 1). The constructed variants’ thermostability was determined by thermally-induced denaturation using DSC (Table 1) and CD spectroscopy (S2A Fig). A substantial increase in melting temperatures (Δ*T*_m_ 16.2°C) was observed for variant DhaA112 designed by the energy-based approach, indicating strong stabilization effects of the introduced mutations (Table 1). The effect of the consensus substitutions was moderate–only two tested variants, DhaA101 and DhaA103, showed positive thermostabilization effects (Δ*T*_m_ 9.6 and 2.3°C), while the mutations in DhaA100 and DhaA102 had neutral or destabilizing effects (Table 1). Combining the best energy-based mutant (DhaA112) with the best mutant identified by the evolution-based approach (DhaA101) produced a final mutant, DhaA115. Effects of the evolution-based substitutions were complementary and additive with those predicted by the energy-based approach, giving an outstanding increase in thermostability: Δ*T*_m_ 24.6°C (Table 1).

#### Characterization of the final combined mutant

**Fig 3:**
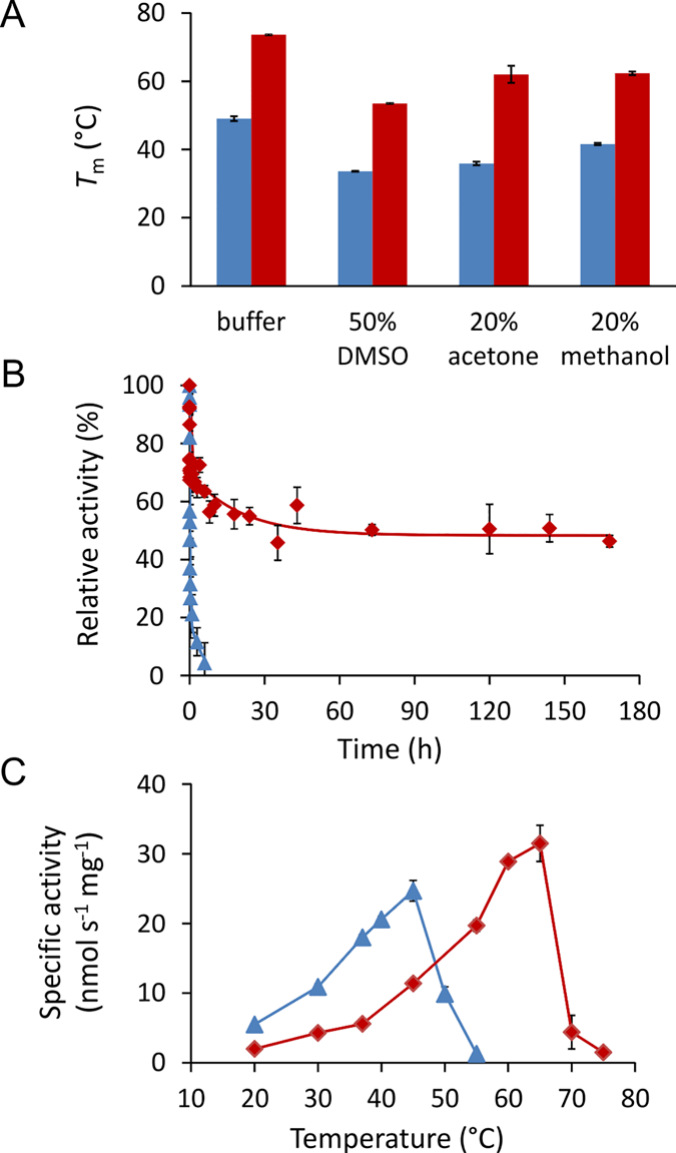
Biochemical properties of DhaA wild-type and the final mutant DhaA115. A) Melting temperatures of DhaA wild-type (blue) and DhaA115 (red) in the presence of indicated solvents. B) Half-life of DhaA wild-type (blue) and DhaA115 (red) determined at 60°C and pH 8.6 with the substrate 1-iodohexane. C) Temperature profiles of DhaA wild-type (blue) and DhaA115 (red) determined at pH 8.6 with the substrate 1-iodohexane.

The combined mutant DhaA115 was characterized in detail, in terms of its thermostability in the presence of organic co-solvents, half-life at elevated temperature and temperature profile. The *T*_m_ determinations show that the mutations had stabilizing effects in the presence of three organic co-solvents comparable to those in the pure buffer (Δ*T*_m_: 20 to 26°C; Fig 3A). The enhanced thermostability was also reflected in a strongly improved half-life at 60°C (Fig 3B). After seven days incubation at 60°C, the mutant DhaA115 still retained about 50% of its initial activity, while the wild-type became inactivated within six hours. Two inactivation phases were observed for all DhaA variants: rapid initial inactivation followed by a slower decay of activity (Fig 3B). The wild-type lost around 80% of its activity in the first fast phase, while the mutations in DhaA115 reduced the loss during the first inactivation phase to only 30%. The rate of inactivation during the first phase was comparable for both wild-type and DhaA115, while the rate during the second phase was dramatically (100-fold) slower for the mutant. Similar effects were observed with a previously reported stable variant of DhaA63 (denoted Dhla8) [28] constructed using Gene Site-Saturation Mutagenesis (S3 Fig). The apparent optimal temperature shifted from 45°C for the wild-type enzyme to 65°C for the mutant DhaA115, and its specific activity with 1-iodohexane at optimum temperature was 28% higher, but the shape of the temperature profile remained largely unchanged (Fig 3C). Steady-state kinetic constants of the two enzymes determined at their respective suboptimal temperatures with 1-iodohexane revealed comparable catalytic properties (Table 2).

**Table 2:** Steady-state kinetic constants of DhaA wild-type and the final mutant Dha115 determined with 1-iodohexane at 37°C and 57°C, respectively, and pH 8.6.

Enzyme	*k*_cat_ (s^-1^)	*K*_0.5_ (μM)	*n*	*K*_si_ (mM)
DhaAwt	2.47 ± 0.01	12.00 ± 1.14	1.80 ± 0.04	0.53 ± 0.01
DhaA115	2.85 ± 0.03	5.00 ± 2.31	1.89 ± 0.01	-

*K*_0.5_ –concentration of substrate at half maximal velocity, *k*_cat_−catalytic constant, *n*–Hill coefficient *K*_si_−substrate inhibition constant

### Design of thermostable γ-hexachlorocyclohexane dehydrochlorinase LinA

γ-hexachlorocyclohexane dehydrochlorinase LinA enzyme was selected as the second model protein to illustrate broader applicability of FireProt strategy to other proteins of very different characteristics: (i) LinA is natively homotrimer (DhaA is monomer), (ii) LinA monomers form α+β barrel fold (DhaA possesses α/β-hydrolase fold), (iii) LinA is mainly composed of β-sheets (α-helices and β-sheets and equally represented in DhaA) and (iv) LinA is with 156 amino acids two-times shorter (DhaA has 294 residues).

#### Energy-based approach

Out of 2,689 possible single-point mutations, 1,533 passed the evolutionary conservation or correlation filter. FoldX analysis of ΔΔG for the remaining mutations identified 68 potentially stabilizing single-point mutations. Subsequent Rosetta calculation further decreased the number of promising mutations to 8 (S9 Table). A mutation D19M disrupting a salt bridge and T133L with antagonistic effect with residue D3 were also discarded, leaving 6 potentially stabilizing mutations at four positions. Only the most favorable mutations at each position were further analyzed: D3I, S127Y, T133I and A145H (Fig 3B). The Rosetta predicted high stability of the recombined multiple-point mutant LinA01 carrying all these four mutations (Table 3).

**Table 3:** Characteristics of predicted multiple-point mutants of LinA.

Method	Protein	Mutations	Rosetta ΔΔG (kcal.mol^-1^)	DSC		Activity at 30°C ^b^	
				*T*_m_ (°C)	Δ*T*_m_ (°C)	(μmol s^-1^ mg^-1^)	
	LinAwt	-^a^	-^a^	41.4 ± 0.1	-^a^	0.21 (0.12 mM)^c^	1.91 (0.38 mM)^c^
Energy-based	LinA01	D3I + S127Y + T133I + A145H	-31.4	62.3 ± 0.4	+20.9	0.32 (0.12 mM)^c^	1.29 (0.34 mM)^c^
Evolution-based	LinA02	Y50F + F68W + A131V	0.1	37.7 ± 0.2	-3.7	0.17 (0.11 mM)^c^	ND

^a^ not applicable

^b^ activity determined with γ-hexachlorocyclohexane at 30°C and pH 8.6

^c^ initial γ-HCH concentration is given since it affects determined specific activity; ΔΔG–predicted change in Free Gibbs Energy; DSC–Differential Scanning Calorimetry; ND, not determined

#### Evolution-based approach

The back-to-consensus identified 15 potentially stabilizing substitutions (S10 Table). Of these, 10 were excluded as destabilizing due to FoldX predictions. Mutation K20Y touches the halide-stabilizing residue and F113Y has a negative effect on enzyme activity according to Uniprot database. Remaining 3 back-to-consensus mutations passed all of the applied filters and were combined into three-point mutant: Y50F, F68W and A131V (Fig 3B).

#### Characterization of mutants designed by FireProt

Expression and purification of all constructed mutants resulted in good protein yields and purity. Comparison of the far-UV CD spectra of LinA wild-type and its mutants show that none of the mutations caused significant changes in secondary structure (S2B Fig). Both LinA variants retained similar level of specific activity as LinAwt (Table 3) showing that the introduced mutations did not alter activity negatively. The thermostability of the constructed LinA variants was determined by thermally-induced denaturation using both DSC (Table 3) and CD spectroscopy (S2B Fig). Similar to the energy-based DhaA variant, the energy-based LinA variant (LinA01) showed a substantial increase in melting temperatures (Δ*T*_m_ 20.9°C) again showing the strong stabilization effects of the introduced mutations (Table 3). The evolution-based mutant (LinA02) showed small decrease in thermostability (Δ*T*_m_ -3.7°C) indicating that mutations identified by consensus methods are not conserved to preserve the stability of the enzyme (Table 3). No combined mutant was constructed due to the absence of stable evolution-based mutations.

### Structural basis of mutation effect

Visual inspection of mutant structures coupled with detailed analysis of their individual energy terms calculated by Rosetta provided indications of the possible structural basis of protein stabilization by mutations of DhaA115 and LinA01 (S11 Table). These mutations were introduced to various locations in the protein structure with different types of secondary structures.

#### Stabilizing mutations in DhaA

Out of 11 mutations, 3 residues are lining a main transport tunnel, 3 mutated residues were buried in the protein core and 5 are exposed to solvent on the protein surface (Fig 2A and S11 Table). 8 mutations identified by the energy-based approach introduce bulkier, more hydrophobic residues (S11 Table) that probably enhance stability by improving packing of atoms in the protein interior and/or strengthening hydrophobic interactions. The contributions to stability of the mutations proposed by the evolution-based approach are more difficult to explain. The A155P mutation (at the fourth most flexible position in the protein structure) could increase rigidity by introducing proline to the affected loop and account for most of the observed stability improvement, while effects of the E20S and F80R mutations are probably neutral or restructuring the charged network on the surface of DhaA (S11 Table).

#### Stabilizing mutations in LinA

Out of 4 stabilizing mutations, 2 are buried in the protein core and 2 are exposed on the protein surface (Fig 2B and S11 Table). In correspondence with observation for stabilizing mutations introduced into DhaA enzyme, all 4 mutations identified by the energy-based approach for LinA introduced bulkier and more hydrophobic residues (S11 Table).

## Discussion

**Fig 4:**
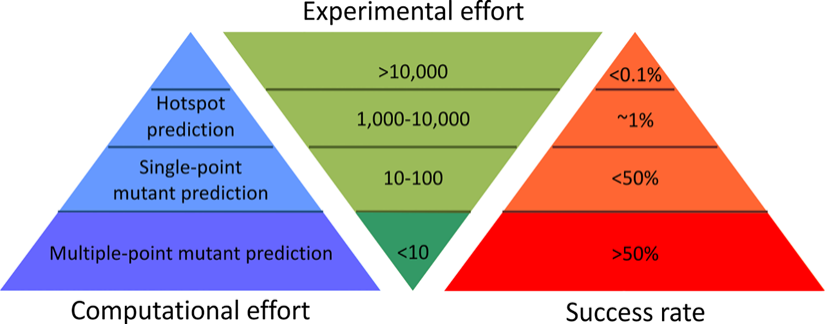
Schematic comparison of protein stabilization methods. Examples of representative methods with their characteristics and success rates are presented in **S12 Table**.

The last decade has seen significant advances towards more rational approaches to reduce the experimental effort required to engineer highly stable proteins (Fig 4 and S12 Table). As a contribution to these efforts we have developed a hybrid strategy integrating energy-based and evolution-based approaches, with smart filtering of mutations that are destabilizing or may impair enzymes’ functions, enabling the identification of additively stabilizing substitutions in multiple-point mutants. It is essential to correctly configure all of the tools used in both the energy- and evolution-based approaches of the FireProt workflow in order to achieve robust and reliable predictions. Therefore, individual steps of the workflow were verified using a dataset featuring diverse proteins from the ProTherm database. The predictions carried out for 656 mutations confirmed the FireProt's precision: the energy- and evolution-based approaches identified stabilizing mutations with success rates of 100% and 80%, respectively. Strikingly, only one stabilizing mutation that exceeded our thresholds was identified by both approaches, suggesting that they are highly complementary. The potential downside of the stringent conditions imposed to avoid false positives was that 92% of the available stabilizing mutations were discarded. However, the remaining correctly identified stabilizing mutations should be more than sufficient to construct highly stable catalysts (S3 Table).

When the energy-based approach was applied to DhaA and LinA enzymes, the removal of conserved and correlated positions from analysis helped to avoid modification of structurally and functionally important residues, thereby greatly reducing the number of possible mutations requiring evaluation by computationally intensive free energy calculation. Since FoldX computation is about an order of magnitude faster than Rosetta, it was applied as a pre-filter, further reducing numbers of mutations to be analyzed by Rosetta. Regarding the prediction of multiple-point mutants, simple recombination of the most promising mutants could weaken stabilization, since strong antagonistic effects were detected even at the level of double-point mutants. The thermostability enhancement for the eight- and four-point mutants predicted by this approach, DhaA112 (Δ*T*_m_ 16°C) and LinA01 (Δ*T*_m_ 21°C), both exceeded the threshold for outstanding stabilization, although none of the introduced mutations optimized either hydrogen bonds or charge-charge interactions. This may be due to sampling limited rotamer libraries during the calculations and the requirement for both FoldX and Rosetta to unambiguously evaluate selected mutations as stabilizing. FoldX and Rosetta employ simplified scoring functions and despite using three protein structures for analysis, only limited protein flexibility is allowed, implying that it should be possible to supplement mutations proposed by free energy calculations with beneficial substitutions identified using different principles.

To this end, additional mutations were selected by the evolution-based approach. The mutations predicted by the back-to-consensus method were filtered by FoldX to discard mutations proposed due to function-related evolutionary constraints rather than structural stabilization. This filtering step proved to be very important as over half of the mutations were discarded as potentially destabilizing. Interestingly, all five multiple-point mutants DhaA100-DhaA103 and LinA02 were predicted as destabilizing by Rosetta and had to be tested experimentally. While this prediction was accurate for three of them (DhaA100, DhaA102 and LinA02), the other two mutants (DhaA101 and DhaA103) were clearly more stable than the wild-type. This result suggests that some underlying principles important for stability detected by the back-to-consensus method are not captured by the applied Rosetta protocol. We speculate that these may include larger backbone rearrangements, interactions with ions present in the solvent, or other entropic contributions that are not well accounted for in the current protocols. Experimental characterization of these mutants by microcalorimetry, temperature-jump stopped-flow and protein crystallography is currently on-going in our laboratory. Despite its lower reliability, the evolution-based approach should still be considered as useful supplement to the energy-based approach, potentially enabling further improvement in the stability of designed proteins. The final 11-point mutant DhaA115 arising from this hybrid prediction strategy is one of the most stable HLD protein known to date (Δ*T*_m_ > 24°C) [13, 30].

We have compared our strategy against several methods providing exceptional protein stabilization (S12 Table). The experimentally intensive protocols of directed evolution and hot-spot predictions can provide engineered enzymes with comparable enhancement. However, since their success rate is generally below 1%, stable proteins can only be obtained after extensive screening. Notably, two of these studies also focused on improving stability of the enzyme DhaA. In one, an eight-point mutant DhaA was obtained with a Δ*T*_m_ of 18°C after screening all 121,000 possible variants [28]. We have obtained a clearly superior enzyme after experimental evaluation of as few as six mutants, highlighting the importance of removing mutations with antagonistic and uncertain stabilizing effects. In the other study performed with DhaA, four hotspots in an access tunnel were experimentally randomized, requiring experimental screening of 5,000 mutations [13], and the Δ*T*_m_ for the best four-point mutant was 19°C.

Highly stable proteins have been obtained by *in silico* prediction of stabilizing effects of single-point mutations in four recently published studies [15, 31, 32, 33]. In one, 67 variants of epoxide hydrolase with mutations identified as potentially stabilizing by the FRESCO method were experimentally tested, 24 were reportedly more stable than the parent protein, and the variant with the best permutation of mutations had remarkably enhanced thermostability (Δ*T*_m_ 36°C) [15]. Much of this enhancement arose from disulfide bridges at the dimer interface, making this approach particularly suitable for multimeric proteins. In another of the studies, four out of six engineered methionine aminopeptidases designed by the Rosetta_VIP_ method were found to be stabilizing and a combined five-point mutant reportedly had a Δ*T*_m_ of 18°C [31]. The authors noted that their final construct is still less stable than the most thermostable native aminopeptidases and that the method is particularly effective for mutagenesis of buried residues around internal cavities. In the other study, a 12-point mutant of Tobacco 5-epi-aristolochene synthase was generated using the SCADS method with an impressive Δ*T*_m_ (45°C), but at the expense of 98% of catalytic activity at the optimal temperature [32]. In comparison to the methods applied in these and other relevant studies (S12 Table), FireProt affords a reduction of experimental screening effort due to robust identification of stabilizing mutations and ensuring their additivity. In addition, it has promising applicability to diverse proteins, potentially all proteins with known tertiary structure and homologous sequences, due to the diverse locations of introduced mutations and universal applicability of underlying principles.

In summary, the presented hybrid strategy FireProt affords rapid design of stable proteins. Consideration of the additivity of identified potentially beneficial mutations enables prediction of multiple-point mutants with significantly enhanced stability. Despite a dramatic reduction in experimental effort, the workflow provided two proteins with outstanding stability. One of them a HLD with greater thermostability than all known HLD enzymes, either obtained from thermophilic organisms or engineered using extensive combinatorial screening. Furthermore, owing to the smart filtering, this strategy is affordable by users with limited access to powerful computer facilities. In addition, implementation of the FireProt strategy in the web-based protein engineering tool Hotspot Wizard [34] is currently on-going in our laboratory to ensure user convenience.

## Methods

### Bioinformatics analysis

#### Construction of multiple sequence alignments

Sequences of six experimentally characterized HLDs–DhaA, LinB, DrbA, DmbC, DhlA and DmbB–or the sequence of LinA were used as queries for PSI-BLAST [35] searches against the nr NCBI database (version July-2009 and May-2015, respectively) [36], with threshold *E*-values of 10^−10^ and 10^−15^ for the initial BLAST search and inclusion of a sequence in the position-specific matrix, respectively. Sequences collected after three PSI-BLAST iterations were clustered by CD-HIT [37] at 90% identity threshold. The resulting dataset (including 8,226 sequences for DhaA and 946 for LinA) was clustered with CLANS [38] using default parameters and varying threshold *P*-values. Sequences clustering with query at the *P-*value of 10^−29^ were extracted and aligned with MUSCLE [39]. All artificial, incomplete or divergent sequences were removed. Final multiple sequence alignment (MSA) of LinA contained 13 sequences. The prepared MSA of the HLD protein family comprised 168 sequences. All sequences (47) belonging to the HLD-II subfamily were then extracted to create a HLD-II subfamily dataset and aligned with MUSCLE. To reduce possible bias toward highly similar sequences, UniqueProt [40] was used (with a HSSP cut-off value of 40) to select representative sequences from both datasets. The created representative HLD family and HLD-II subfamily datasets comprised 87 and 27 sequences, respectively. Each representative dataset was then aligned with MUSCLE [39].

#### Analysis of evolutionary conservation and correlation

The MSA of the whole HLD-II subfamily or the MSA of LinA was used to estimate the level of conservation at individual positions. Normalized evolutionary rates for each amino acid site of the MSA were calculated by the Bayesian method implemented in Rate4Site v2.01 [24] with the WAG evolutionary model [41] and maximum likelihood optimization of branch lengths using a gamma model with four discrete categories. Calculated normalized evolutionary rates were converted to CONSURF conservation grades [25, 26]. Positions with a grade ≥ 8 were considered immutable. The MSA of the whole HLD family or MSA of LinA was used to identify positions with correlated mutations (and a threshold Correlated Mutation Analysis score ≥ 0.8), by applying the 3DM database’s Comulator online tool [29].

#### Back-to-consensus analysis

Back-to-consensus mutations were selected by analyzing both representative MSAs of HLDs and the MSA of LinA by simple consensus and frequency ratio approaches [27]. Residues from poorly aligned regions (DhaA residues 1–18, 131–179 and 279–293 from the HLD family MSA, and residues 1–14 from the HLD-II subfamily MSA) were excluded from the analysis. The simple consensus analysis was performed using the consensus cut-off of 0.5, meaning that a given residue must be present at a given position in at least 50% of all analyzed sequences to be assigned as the consensus residue. Two cut-offs were simultaneously applied in the frequency ratio analyses: a frequency cut-off of 0.2 for the maximum allowed ratio between target and conserved residue frequencies, and a minimal frequency of 0.4 for the most conserved residue.

#### Compilation of a validation dataset

The validation dataset was compiled from ProTherm records that include experimentally determined differences between the Gibbs free energies of folding for mutant proteins and the corresponding wild type (ΔΔG). To increase the reliability of the analysis, only records with absolute ΔΔG values of ≥ 0.5 kcal.mol^-1^ were included; the experimental error for measurements of ΔΔG is estimated to be about 0.48 kcal.mol^-1^ [19]. When multiple ΔΔG values were available for a single mutation, only the value determined under the experimental conditions closest to the physiological pH of 7 was retained. The dataset was also limited to mutations in the ten most mutated proteins in ProTherm. Multiple sequence alignments for each protein in the dataset were constructed using a protocol similar to that applied in the analysis of the model enzyme DhaA. However, an automatic multi-step procedure was developed to circumvent the need to manually select PSI-BLAST queries. First, the name of the relevant protein family was found in the SCOP database [42]. Then, all members of the same protein family were clustered by CD-HIT using an identity threshold of 90%. Finally, up to five representatives of each resulting cluster were selected at random to constitute the set of PSI-BLAST queries.

### Molecular modeling

#### Preparation of protein structures

Crystal structures of wild-type DhaA (PDB ID: 1BN6, 1BN7 and 1CQW) and wild-type LinA (PDB ID: 3A76) were downloaded from the RCSB PDB database [43]. PyMOL v1.4 [44] was used to model substitutions A172V, I209L and G292A in the crystal structures of DhaA to ensure their correspondence with DhaA from *Rhodococcus rhodochrous* (GI number 3114657). The crystal structures were then prepared for predictions by removing ligands and water molecules. Missing side-chain atoms were added by the <RepairPDB> module of FoldX 3.0 [20]. Repaired structures were minimized by the minimize_with_cst module of Rosetta [21], with: both backbone and side-chains optimization enabled (—*sc_min_only false*), distance for full atom pair potential set to 9 Å (—*fa_max_dis 9*.*0*), standard weights for the score function and a constraint weight of 1 (*—constraint_weight 1*.*0*). Output from the minimization was used to create a constraint file by script convert_to_cst_file.sh.

#### Prediction of stability effects by FoldX

Stability effects of all possible single-point mutations were estimated using the <BuildModel> module of FoldX. Calculations were performed five times for each mutation following the recommended protocol (pH 7, temperature 298 K, ion strength 0.050 M, VdWDesign 2). Mutations with predicted ΔΔG averaged over all three analyzed structures smaller than -1.0 kcal.mol^-1^ were considered as stabilizing, while a tighter criterion was applied for detecting potentially destabilizing mutations (ΔΔG > 0.5 kcal.mol^-1^).

#### Prediction of stability effects by Rosetta

Protocol 16 incorporating backbone flexibility within the ddg_monomer module of Rosetta was applied according to Kellogg et al. [21]. The soft-repulsive design energy function (—*soft_rep_design weights*) was used for repacking side-chains (*—sc_min_only false*). Optimization was performed on each whole protein without distance restriction (—*local_opt_only false*). The previously created constraint file was used during backbone minimization (—*min_cst true*). Three rounds of optimization with increasing weight on the repulsive term (—*ramp_repulsive true*) were applied. The minimum energies from 20 and 50 iterations were used as the final parameters describing the stability effects of single- and multiple-point mutations, respectively. Mutations with ΔΔG averaged over all three analyzed DhaA structures or three chains of LinA < -2.0 kcal.mol^-1^ were considered as stabilizing. The additivity of stabilizing mutations was evaluated by predicting the stability of variants with all pairs of potentially stabilizing single-point mutations. Mutation pairs for which the respective double-point mutants showed predicted ΔΔG > -3.0 kcal.mol^-1^ were considered as antagonistic. The cumulative mutants were then prepared by combining mutually additive single-point mutations starting with the most stabilizing mutation. If there were more than one stabilizing mutations at the same position, the most favorable mutation was used.

#### Analyses of interactions

Selected mutations were visually analyzed in PyMOL. All three crystal structures of DhaA and three chains of LinA were analyzed for the presence of side-chains involved in intra-molecular salt bridges by the ESBRI server [45] and additional intra-molecular interactions using the Protein Interactions Calculator server [46]. An interaction had to be present in at least one of the analyzed structures or chains to be considered as important.

### Construction of mutants and biochemical characterization

#### Subcloning

All reagents and primers were purchased from Sigma-Aldrich unless otherwise specified. Restriction enzymes, T4 DNA ligase and accompanying buffers were purchased from New England Biolabs. Genes encoding tested *Rhodococcus rhodochrous* DhaA (Uniprot: P0A3G2) mutants were cloned in the pET21b (EMD Biosciences) (DhaAwt, 101, 110–112, 115–116) or pAQN vector [47] (DhaA63, 100–103) using the restriction enzyme pairs *Nde*I/*Hind*III or *Bam*HI/*Hind*III, respectively, followed by ligation with T4 DNA ligase according to the supplier’s protocol. The plasmid pET28-LinAwt encoding the wild-type LinA from *Sphingobium japonicum* UT26 (Uniprot: P51697) was a gift from Dr. Yuji Nagata [48]. The genes encoding for the mutants were cloned in the pET28b (EMD Biosciences) using the restriction enzymes *Nde*I and *Eco*RI, followed by ligation with T4 DNA ligase (Promega) according to the supplier’s protocol. Correct integration of the genes was verified by sequencing (GATC Biotech) and analyzed using Clone Manager Professional (Sci-Ed Software) and BioEdit (Ibis Biosciences) software. DhaA and LinA variants were expressed with a C-terminal and N-terminal His_6_-tag, respectively, to facilitate purification.

#### Enzyme expression and purification

The His_6_-tagged DhaA and LinA mutants were overexpressed in *Escherichia coli* BL21(DE3) cells as previously described [47]. Proteins were purified using Ni-NTA Superflow Cartridges (Qiagen) and a previously described method [49]. Protein concentration was determined by assays with the Bradford reagent (Sigma–Aldrich). The purity of purified proteins was checked by sodium dodecyl sulfate polyacrylamide gel electrophoresis (SDS-PAGE) followed by Coomassie Brilliant Blue R-250 staining.

#### Enzyme activity assays with DhaA

Reaction mixtures containing 12 μL of substrate in 12 mL 100 mM glycine buffer (pH 8.6) were preheated at 37°C for 30 min, 240 μL of purified enzyme (0.4–1.0 mg.mL^-1^) was added to initiate the reaction, and it was monitored by withdrawing samples at periodic intervals (0–30 min). The samples were immediately mixed with 35% nitric acid to terminate the reaction. The released halide ions were measured spectrophotometrically at 460 nm after reaction with mercuric thiocyanate and ferric ammonium sulfate [50]. Dehalogenating activity was quantified as the rate of halide product formation per unit time. Temperature profiles of DhaAwt and DhaA115 were evaluated by measuring their activity, as described above, at temperatures ranging from 20°C-75°C in three independent replicates. The operational stability was evaluated by measuring residual activity after incubating 1 mL enzyme samples (1.0 mg.mL^-1^) at 60°C in a heat block (Biosan Pst-100 HL).

Residual activity was determined using a Microlab StarLet Manuload Liquid Handling Robot (Hamilton). For the residual activity measurements, 1 mL of 100 mM Glycine buffer (pH 8.6) with 1 μL 1,2-dibromoethane was incubated at 37°C for 30 min then 50 μL of heat-treated enzyme solution was added to start the reaction (DhaAwt 0.1 mg.mL^-1^, DhaA115 and DhaA63 1.0 mg.mL^-1^). Samples (100 μL) were taken before enzyme addition (0 min) and after 5, 10 and 15 min reaction time. Samples were then transferred to wells of a MTP microplate containing 10 μL 35% H_3_NO_4_ for inactivation. After all samples had been collected halide product was detected as described earlier. OD_460nm_ was then measured using a Sunrise microplate reader (Tecan). Dehalogenation activities were quantified by the slope of the regression between the product concentration and time.

#### Enzyme activity assay with LinA

The activity of the LinA variants was tested with γ-hexachlorocyclohexane (γ-HCH) at 30°C and analyzed using GC-MS. Saturating γ-HCH substrate mixtures in 1 mL 100 mM glycine buffer (pH 8.6) were preheated at 30°C for 30 min. 10 μL of purified enzyme (8.9–28.8 μg.mL^-1^) was added to initiate the reaction that was monitored by withdrawing 1 μL samples at 15 min periodic intervals (0–75 min). The samples were immediately analyzed by GC-MS. Gas chromatograph equipped with the PAL robotic tool change system enabled fully automatized sample preparations, organic extractions and analysis. The consumption of particular substrates was quantified using gas chromatography (Trace 1300, Thermo Scientific, USA) equipped with capillary column TG-SQC (30m x 0.25mm x 0.25μm, Thermo Scientific, USA) and connected with mass spectrometer (ISQ™ LT Single Quadrupole, Thermo Scientific, USA). The 1 μL samples were injected into split-splitless inlet at 250°C, with split ratio 1:50. The temperature program was isothermal at 40°C for 1 min, followed by increase to 250°C at 20°C.min^-1^ and hold for 4 min. The flow of carrier gas (He) was 1 mL.min^-1^. The MS was operated at SCAN mode (30 to 320 amu). The temperature of ion source and GC-MS transfer line was 200°C and 250° C, respectively. Dehydrochlorination activities were quantified following the decrease in concentration of γ-HCH over time.

#### Steady-state kinetic measurements

Substrate to product conversion by the action of DhaAwt and DhaA115 was monitored by using the isothermal titration microcalorimeter VP-ITC (MicroCal, Piscataway, USA) at 37°C and 57°C, respectively. The substrate 1-iododohexane was dissolved in 100 mM glycine buffer (pH 8.6) and the solution was allowed to reach thermal equilibrium in the reaction cell (1.4 mL). The reaction was initiated by injecting 10 μL of enzyme solution containing either 22 μM DhaAwt or 24 μM DhaA115 into the reaction cell. Enzymes were dialyzed overnight against the same glycine buffer. The measured rate of heat change was assumed to be directly proportional to the velocity of the enzymatic reaction according to the Eq (1)

$$\frac{dQ}{dt}=-\Delta HV\frac{d[S]}{dt}$$ (1)

where *ΔH* is the enthalpy of the reaction, [S] is the substrate concentration, and *V* is the volume of the cell. *ΔH* was determined by titrating the substrate into the reaction cell containing the enzyme. Each reaction was allowed to proceed to completion. The integrated total heat of a reaction was divided by the amount of injected substrate. The evaluated rate of substrate depletion (-*d*[*S*]/*dt*) and corresponding substrate concentrations were then fitted by nonlinear regression to kinetic models using Origin 6.1 (OriginLab, Massachusetts, USA).

#### Circular dichroism (CD) spectroscopy

CD spectra (190 to 260 nm) were obtained from samples of the purified enzymes (0.20–0.25 mg.mL^-1^ in 50 mM phosphate buffer, pH 7.5, in a 0.1 cm quartz cuvette) to confirm their correct folding, using a Chirascan CD Spectrometer equipped with a Peltier thermostat (Applied Photophysics). Each presented spectrum is the baseline-corrected average of 5–10 scans. Mean residue ellipticity (Θ_MRE_) was calculated using Eq (2)

$$\Theta_{\text{MRE}}=\frac{\Theta_{\text{obs}}.M_{\text{w}}.100}{n.c.l}$$ (2)

where Θ_obs_ is observed ellipticity in degrees, M_*w*_ is the protein molecular weight, *n* is number of residues, *l* is the cell path length (0.1 cm), *c* is the protein concentration and the factor 100 originates from conversion of the molecular weight to mg.dmol^-1^ [51].

Thermal unfolding of the enzyme variants was followed by monitoring the ellipticity at 222 nm over the temperature range of 20°C to 90°C, with a resolution 0.2°C, at a heating rate at 1°C/min. Recorded denaturation curves of tested enzyme were fitted to sigmoid curves (Boltzmann) using OriginPro8 software (OriginLab, Massachusetts, USA). The melting temperatures (*T*_m_) were evaluated from the collected data as a midpoint (x_0_) of the normalized thermal transition.

#### Differential scanning calorimetry (DSC)

Melting temperatures of the purified enzymes were determined by monitoring their heat capacity in solution (1.0 mg.mL^-1^) in 50 mM aqueous phosphate buffer (pH 7.5) and in the presence of three cosolvents: 20% acetone, 20% methanol and 50% DMSO (v/v). The measurements were acquired, after degassing, at temperatures from 20 to 100°C using the VP-capillary differential scanning calorimetry (DSC) system (MicroCal) and a 1°C.min^-1^ heating rate. The melting point of each protein was determined as the temperature at which the heat capacity curve peaked [52].

## Supporting Information

S1 Fig Correlation between ΔTm and ΔΔG values of 46 DhaA mutants. The experimentally characterized homogenous set of DhaA mutants [1, 2] employed during validation of the Rosetta approach is shown as blue squares. The red line represents the trend in the experimentally characterized mutants (correlation coefficient, 0.81).
(TIF)

S2 Fig Far-UV CD spectra of the tested mutants and determined melting temperatures. A) Variants of haloalkane dehalogenase DhaA. B) Variants of γ-hexachlorocyclohexane dehydrochlorinase LinA. The melting temperatures (*T*_m_) were evaluated as midpoints of the normalized thermal transitions.
(TIF)

S3 Fig Half-life of DhaA63 determined at 60°C in 50 mM phosphate buffer pH 7.5. (TIF)

S1 Table Composition of single-point mutation dataset derived from ProTherm database. (PDF)

S2 Table Performance of four evaluated prediction tools at different decision thresholds. (PDF)

S3 Table Stabilizing mutations selected for the 10 most mutated proteins from ProTherm. (PDF)

S4 Table Results of the energy-based analysis of DhaA. (PDF)

S5 Table Results of the frequency ratio analysis of the HLD-II subfamily. (PDF)

S6 Table Results of the simple consensus analysis of the HLD-II subfamily. (PDF)

S7 Table Results of the frequency ratio analysis of the HLD family. (PDF)

S8 Table Results of the simple consensus analysis of the HLD family. (PDF)

S9 Table Results of the energy-based analysis of LinA. (PDF)

S10 Table Results of the consensus analysis of LinA. (PDF)

S11 Table Predicted effects of DhaA115 and LinA01 mutations on its stability. (PDF)

S12 Table Examples of methods providing enzymes with outstanding stabilization. (PDF)

S1 Text Supporting references. (PDF)
