# Supplementary figures and images for: FireProt: Energy- and Evolution-Based Computational Design of Thermostable Multiple-Point Mutants
Source: PLoS Comput Biol. 2015 Nov 3;11(11):e1004556. doi: 10.1371/journal.pcbi.1004556 (PMC4631455; doi:10.1371/journal.pcbi.1004556)

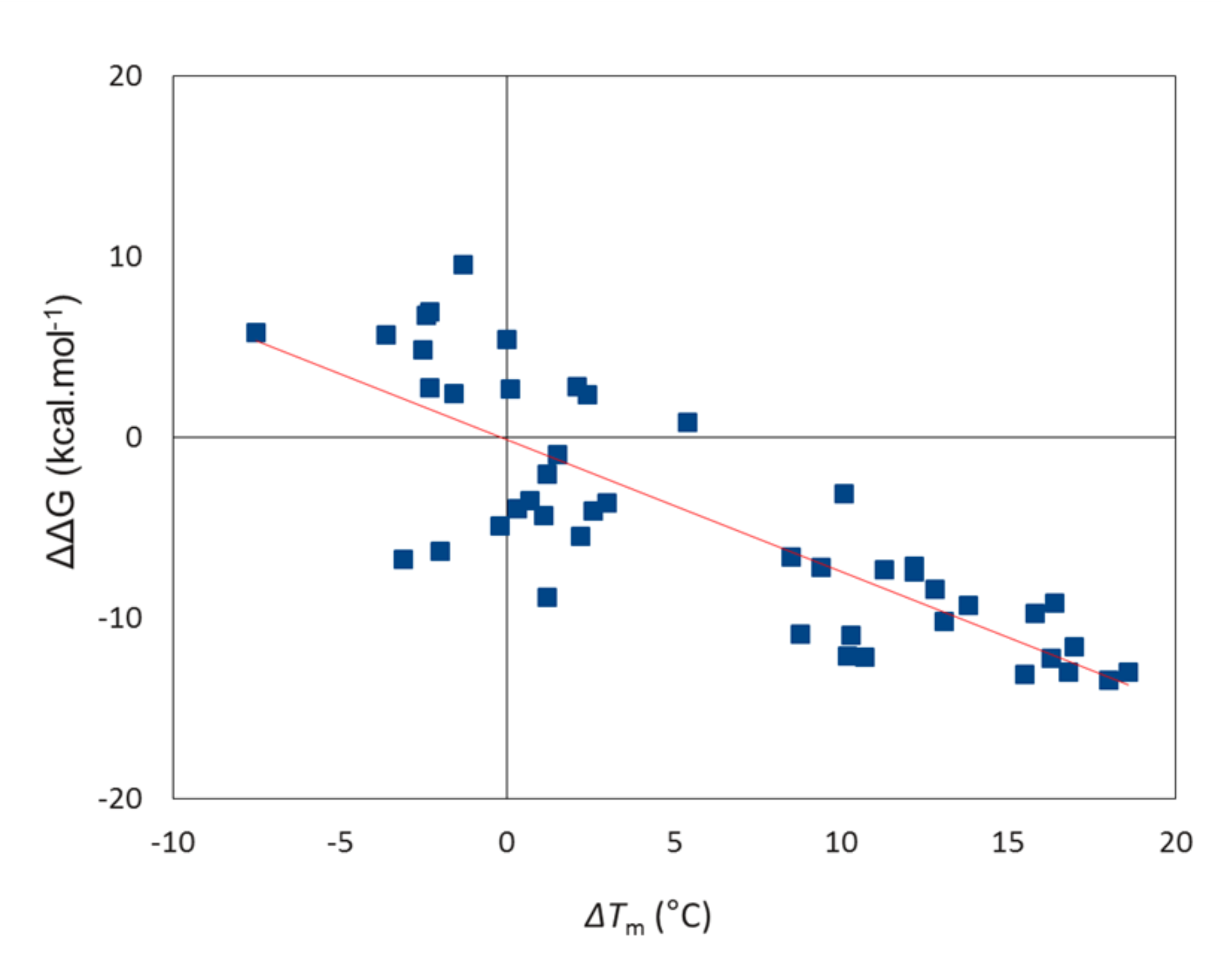

Supplement: S1 Fig — The experimentally characterized homogenous set of DhaA mutants [1, 2] employed during validation of the Rosetta approach is shown as blue squares. The red line represents the trend in the experimentally characterized mutants (correlation coefficient, 0.81). (TIF) [file pcbi.1004556.s001.tif]

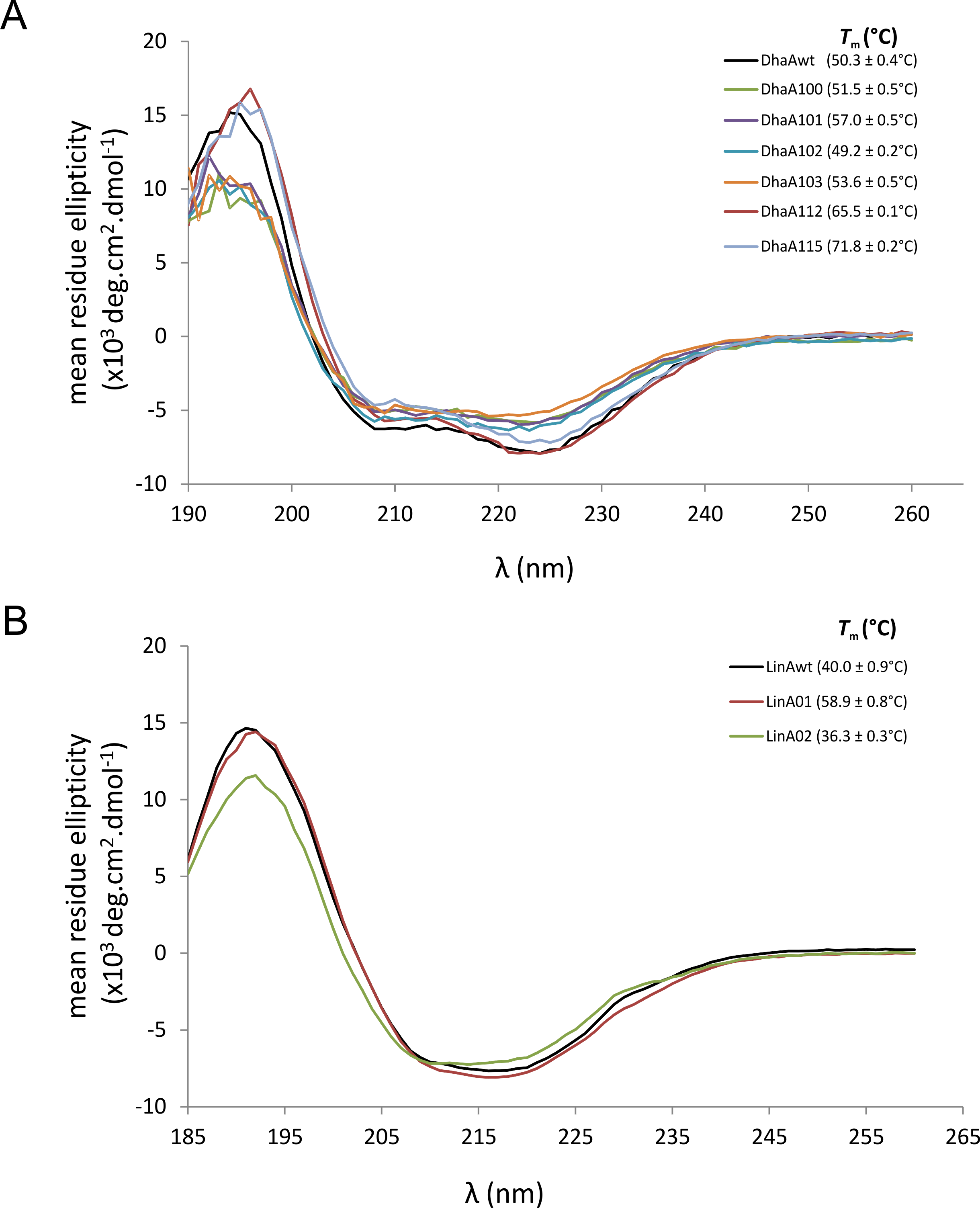

Supplement: S2 Fig — A) Variants of haloalkane dehalogenase DhaA. B) Variants of γ-hexachlorocyclohexane dehydrochlorinase LinA. The melting temperatures (T m) were evaluated as midpoints of the normalized thermal transitions. (TIF) [file pcbi.1004556.s002.tif]

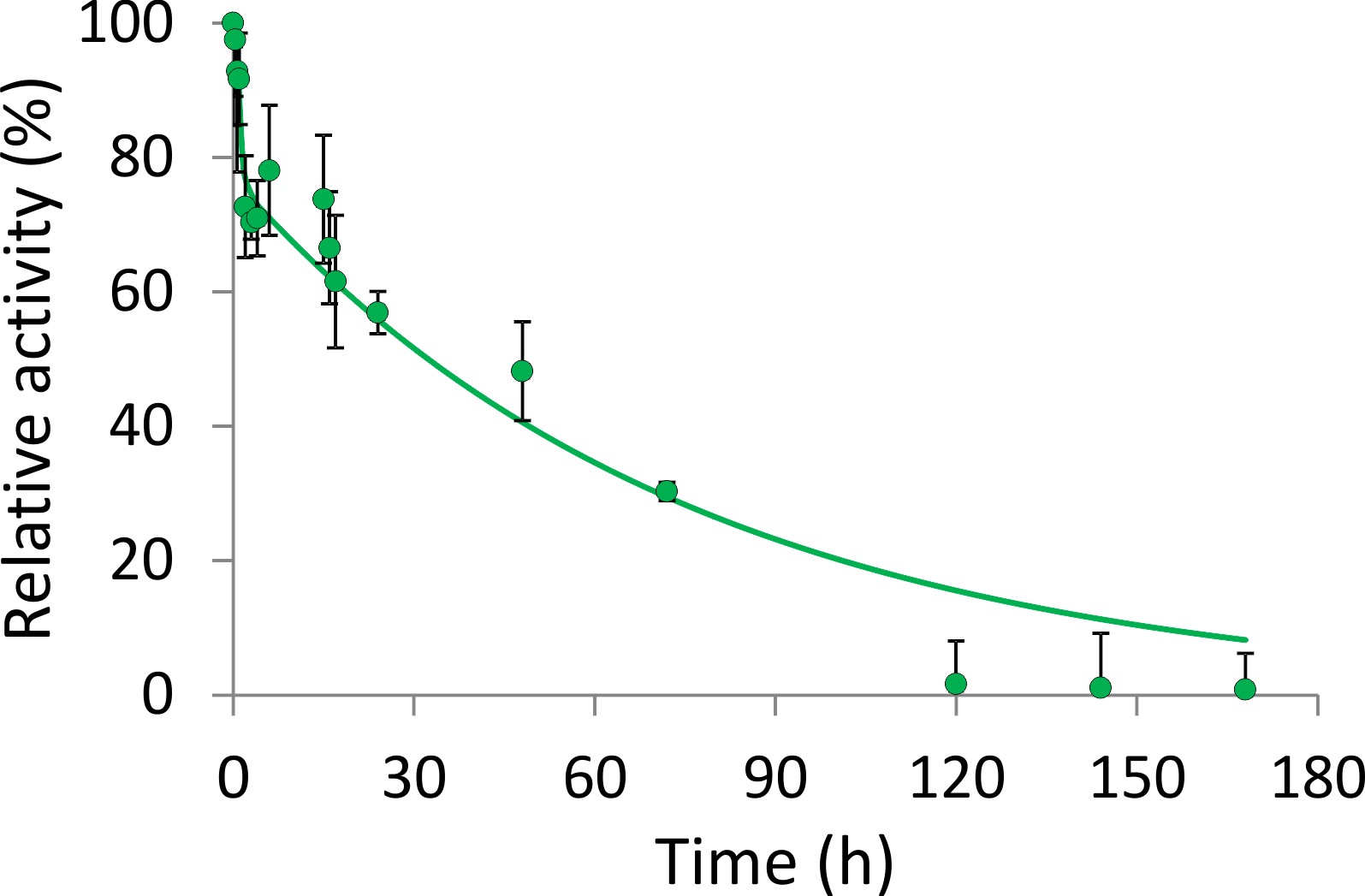

Supplement: S3 Fig — (TIF) [file pcbi.1004556.s003.tif]
