# Supplementary material for: FireProt: Energy- and Evolution-Based Computational Design of Thermostable Multiple-Point Mutants
Source: PLoS Comput Biol. 2015 Nov 3;11(11):e1004556. doi: 10.1371/journal.pcbi.1004556 (PMC4631455; doi:10.1371/journal.pcbi.1004556)
Supplement: S1 Table — (PDF) [file pcbi.1004556.s004.pdf]

S1 Table. Composition of single-point mutation dataset derived from ProTherm database.

PDB ID	Protein	Organism	Structural class	Number of mutations			Number of positions
				Total	Stabilizing	Destabilizing	
2LZM	Lysozyme	Bacteriophage T4	α+β	155	25	130	80
1BNI	Barnase	*Bacillus amyloliquefaciens*	α+β	124	4	120	54
1LZ1	Lysozyme	Human	α+β	85	19	66	42
1VQB	Gene V	Bacteriophage f1	all β	60	6	54	24
2CI2	Chymotrypsin inhibitor	Barley	α+β	56	3	53	39
1CSP	Cold shock protein	*Bacillus subtilis*	all β	40	20	20	24
2RN2	Ribonuclease HI	*Escherichia coli*	α/β	38	21	17	12
1BVC	Myoglobin	Sperm whale	all α	36	5	31	21
1RN1	Ribonuclease T1	*Aspergillus oryzae*	α+β	33	6	27	23
4LYZ	Lysozyme	Chicken	α+β	29	10	19	18
