# Supplementary material for: FireProt: Energy- and Evolution-Based Computational Design of Thermostable Multiple-Point Mutants
Source: PLoS Comput Biol. 2015 Nov 3;11(11):e1004556. doi: 10.1371/journal.pcbi.1004556 (PMC4631455; doi:10.1371/journal.pcbi.1004556)
Supplement: S2 Table — (PDF) [file pcbi.1004556.s005.pdf]

S2 Table. Performance of four evaluated prediction tools at different decision thresholds.

Metric	Tool	Decision threshold [kcal/mol]										
		**2.5**	**2.0**	**1.5**	**1.0**	**0.5**	**0.0**	**-0.5**	**-1.0**	**-1.5**	**-2.0**	**-2.5**
**Precision (Ratios)**	FoldX	0.23	0.25	0.27	0.32	0.39	0.51	0.63	0.67	0.60	0.67	0.50
	Rosetta	0.26	0.28	0.31	0.35	0.42	0.49	0.59	0.65	0.71	0.76	0.75
	ERIS	0.29	0.31	0.33	0.34	0.38	0.39	0.40	0.39	0.43	0.36	0.36
	CUPSAT	0.21	0.22	0.24	0.27	0.29	0.29	0.27	0.20	0.14	0.09	0.03
**Precision (Absolute values)**	FoldX	115/510	114/464	112/408	109/341	93/237	77/151	39/62	20/30	9/15	4/6	1/2
	Rosetta	114/438	111/394	108/344	99/281	92/218	74/151	62/105	46/71	25/35	16/21	9/12
	ERIS	97/331	89/285	80/240	67/195	57/151	50/127	37/92	27/70	17/40	10/28	8/22
	CUPSAT	119/569	118/529	116/479	110/415	98/343	77/266	55/206	28/141	12/86	5/55	1/29
**False positive rate (Ratios)**	FoldX	0.74	0.65	0.55	0.32	0.27	0.14	0.04	0.02	0.01	0.00	0.00
	Rosetta	0.60	0.53	0.44	0.35	0.23	0.14	0.08	0.05	0.02	0.01	0.01
	ERIS	0.44	0.37	0.30	0.34	0.18	0.15	0.10	0.08	0.04	0.03	0.03
	CUPSAT	0.84	0.77	0.68	0.57	0.46	0.35	0.28	0.21	0.14	0.09	0.05
**False positive rate (Absolute values)**	FoldX	395/537	350/537	296/537	232/537	144/537	74/537	23/537	10/537	6/537	2/537	1/537
	Rosetta	324/537	283/537	236/537	182/537	126/537	77/537	43/537	25/537	10/537	5/537	3/537
	ERIS	324/537	283/537	236/537	182/537	126/537	77/537	43/537	25/537	10/537	5/537	3/537
	CUPSAT	450/537	411/537	363/537	305/537	245/537	189/537	151/537	113/537	74/537	50/537	28/537

Precision (true positive/(true positive + false positive)) represents the ratio between the truly stabilizing mutations and mutations predicted as stabilizing by a given tool.

False positive rate (false positive/(true negative + false positive)) represents the fraction of destabilizing mutations incorrectly predicted as stabilizing by a given tool from all truly destabilizing mutations.

The threshold possessing the highest precision and at the same time the highest number of true positives for individual tool is highlighted.
