# Supplementary material for: FireProt: Energy- and Evolution-Based Computational Design of Thermostable Multiple-Point Mutants
Source: PLoS Comput Biol. 2015 Nov 3;11(11):e1004556. doi: 10.1371/journal.pcbi.1004556 (PMC4631455; doi:10.1371/journal.pcbi.1004556)
Supplement: S3 Table — (PDF) [file pcbi.1004556.s006.pdf]

S3 Table. Stabilizing mutations selected for the 10 most mutated proteins from ProTherm.

	Number of mutations predicted as stabilizing by individual tool			
**PDB ID**	**Rosetta** [a]	**FoldX** [b]	**Rosetta and FoldX** [c]	**FireProt** [d]
1BVC	105	119	26	23
1LZ1	44	60	5	4
1VQB	74	62	18	7
2LZM	99	106	18	16
4LYZ	150	39	6	5
1BNI	132	118	19	6
1CSP	31	51	4	2
1RN1	97	207	23	18
2CI2	48	37	10	9
2RN2	111	120	25	17
**Total**	**891**	**919**	**154**	**107**

[a] Number of Rosetta predictions with ddG < -2 kcal/mol.

[b] Number of FoldX predictions with ddG < -1 kcal/mol.

[c] Number of predictions with Rosetta ddG < -2 kcal/mol and FoldX ddG < -1 kcal/mol.

[d] Number of predictions identified by FireProt using criteria defined in the Methods.
