# Supplementary material for: FireProt: Energy- and Evolution-Based Computational Design of Thermostable Multiple-Point Mutants
Source: PLoS Comput Biol. 2015 Nov 3;11(11):e1004556. doi: 10.1371/journal.pcbi.1004556 (PMC4631455; doi:10.1371/journal.pcbi.1004556)
Supplement: S4 Table — (PDF) [file pcbi.1004556.s007.pdf]

S4 Table. Results of the energy-based analysis of DhaA.

Position	Residue	Mutation	FoldX ΔΔG (kcal.mol^{-1})	Rosetta ΔΔG (kcal.mol^{-1})	Antagonistic effect	Interactions	Mutant
20	E	Q	-1.09	-2.13	C128F	-	-
128	C	F	-2.21	-8.45	-	-	DhaA112
		M	-3.48	-2.96	-	-	-
148	T	W	-1.09	-2.65	C128F	-	-
		L	-1.96	-2.00	-	-	DhaA112
172	A	V	-1.92	-2.21	C176F	-	-
		I	-2.83	-2.16	-	-	DhaA112
176	C	F	-2.22	-7.07	-	-	DhaA112
		L	-2.01	-5.28	-	-	-
		H	-1.08	-4.82	-	-	-
		M	-2.51	-4.24	-	-	-
187	D	W	-1.37	-2.58	-	R190	-
198	D	W	-1.36	-4.55	-	-	DhaA112
		F	-1.98	-2.95	-	-	-
		Y	-1.85	-2.75	-	-	-
		L	-1.92	-2.53	-	-	-
217	N	Y	-2.38	-2.38	C128F	-	-
219	V	W	-1.77	-3.04	-	-	DhaA112
262	C	L	-1.64	-4.93	-	-	DhaA112
		M	-1.42	-2.94	-	-	-
266	D	Y	-2.43	-2.90	C128F	-	-
		F	-2.31	-2.41	-	-	DhaA112
