# Supplementary material for: FireProt: Energy- and Evolution-Based Computational Design of Thermostable Multiple-Point Mutants
Source: PLoS Comput Biol. 2015 Nov 3;11(11):e1004556. doi: 10.1371/journal.pcbi.1004556 (PMC4631455; doi:10.1371/journal.pcbi.1004556)
Supplement: S5 Table — (PDF) [file pcbi.1004556.s008.pdf]

S5 Table. Results of the frequency ratio analysis of the HLD-II subfamily.

Position	Residue	Frequency	[a]Res_TOP	[b]Freq_TOP	Frequency ratio	FoldX ΔΔG ($kcal.mol^{-1}$)	Interactions	Mutant
20	E	0.07	S	0.44	0.17	0.38	-	DhaA101
59	H	0.11	G	0.56	0.2	2.36	-	-
77	L	0.07	I	0.44	0.17	1.90	-	-
80	F	0.04	R	0.44	0.08	0.37	-	DhaA101
128	C	0.04	F	0.41	0.09	-2.21	L237	-
132	I	0.07	V	0.59	0.12	0.92	-	-
155	A	0.07	P	0.44	0.17	-0.84	-	DhaA101
159	R	0.07	E	0.78	0.1	-0.62	E200	-
163	I	0.07	L	0.7	0.11	-0.37	-	DhaA100
184	V	0.04	E	0.52	0.07	-0.59	-	DhaA100
197	V	0.04	E	0.52	0.07	-0.20	-	DhaA100
200	E	0.04	R	0.59	0.06	-0.59	R159	-
203	W	0.15	L	0.78	0.19	0.67	F152, N207	-
207	N	0.15	R	0.81	0.18	1.88	F152, W203	-
218	I	0.07	V	0.7	0.11	0.56	-	-
267	I	0.11	V	0.63	0.18	0.92	-	-
278	N	0.07	S	0.44	0.17	1.91	I267, L281	-

[a] The most conserved residue at a given position of the multiple sequence alignment; [b] Frequency of the most conserved residue at a given position of the multiple sequence alignment
