# Supplementary material for: FireProt: Energy- and Evolution-Based Computational Design of Thermostable Multiple-Point Mutants
Source: PLoS Comput Biol. 2015 Nov 3;11(11):e1004556. doi: 10.1371/journal.pcbi.1004556 (PMC4631455; doi:10.1371/journal.pcbi.1004556)
Supplement: S6 Table — (PDF) [file pcbi.1004556.s009.pdf]

S6 Table. Results of the simple consensus analysis of the HLD-II subfamily.

Position	Residue	Frequency	[a]Res_TOP	[b]Freq_TOP	FoldX ΔΔG ($kcal.mol^{-1}$)	Interactions	Mutant
36	L	0.26	V	0.59	1.26	-	-
51	I	0.44	V	0.52	0.72	-	-
59	H	0.11	G	0.56	2.36	-	-
93	E	0.3	D	0.52	0.69	R122	-
119	N	0.3	H	0.63	-0.80	W115, R122	-
132	I	0.07	V	0.59	0.92	-	-
159	R	0.07	E	0.78	-0.62	E200	-
161	L	0.22	M	0.59	0.01	-	**DhaA102**
162	I	0.3	V	0.56	0.48	-	**DhaA102**
163	I	0.07	L	0.7	-0.37	-	**DhaA100**
169	I	0.37	V	0.52	0.76	-	-
184	V	0.04	E	0.52	-0.59	-	**DhaA100**
197	V	0.04	E	0.52	-0.20	-	**DhaA100**
198	D	0.19	S	0.56	-0.45	-	**DhaA102**
200	E	0.04	R	0.59	-0.59	R159	-
202	L	0.19	T	0.52	3.07	-	-
203	W	0.15	L	0.78	0.67	F152, N207	-
205	F	0.26	W	0.7	2.30	-	-
207	N	0.15	R	0.81	1.88	F152, W203	-
218	I	0.07	V	0.7	0.56	-	-
241	G	0.15	A	0.67	0.80	-	-
267	I	0.11	V	0.63	0.92	-	-
273	Y	0.15	F	0.67	0.30	N41	-
285	E	0.11	A	0.52	-0.23	K263	-

[a] The most conserved residue at a given position of the multiple sequence alignment; [b] Frequency of the most conserved residue at a given position of the multiple sequence alignment
