# Supplementary material for: FireProt: Energy- and Evolution-Based Computational Design of Thermostable Multiple-Point Mutants
Source: PLoS Comput Biol. 2015 Nov 3;11(11):e1004556. doi: 10.1371/journal.pcbi.1004556 (PMC4631455; doi:10.1371/journal.pcbi.1004556)
Supplement: S7 Table — (PDF) [file pcbi.1004556.s010.pdf]

S7 Table. Results of the frequency ratio analysis of the HLD family.

Position	Residue	Frequency	[a]Res_TOP	[b]Freq_TOP	Frequency ratio	FoldX ΔΔG (kcal.mol^{-1})	Interactions	Mutant
27	V	0.05	E	0.49	0.09	1.42	-	-
188	H	0.07	A	0.51	0.14	-0.04	-	**DhaA103**
191	E	0.1	A	0.55	0.19	0.10	-	**DhaA103**
271	L	0.09	G	0.57	0.16	2.92	-	-

[a] The most conserved residue at a given position of the multiple sequence alignment; [b] Frequency of the most conserved residue at a given position of the multiple sequence alignment
