# Supplementary material for: FireProt: Energy- and Evolution-Based Computational Design of Thermostable Multiple-Point Mutants
Source: PLoS Comput Biol. 2015 Nov 3;11(11):e1004556. doi: 10.1371/journal.pcbi.1004556 (PMC4631455; doi:10.1371/journal.pcbi.1004556)
Supplement: S8 Table — (PDF) [file pcbi.1004556.s011.pdf]

S8 Table. Results of the simple consensus analysis of the HLD family.

Position	Residue	Frequency	[a]Res_TOP	[b]Freq_TOP	FoldX ΔΔG (kcal.mol^{-1})	Interactions	Mutant
44	S	0.3	W	0.69	12.12	Y46	-
55	V	0.16	L	0.53	0.31	-	**DhaA103**
109	S	0.21	G	0.71	2.13	D106. I132	-
111	L	0.37	I	0.55	1.26	-	-
127	A	0.25	V	0.54	-2.32	-	**DhaA103**
130	E	0.3	N	0.67	0.21	V245. L246. I247. L271. H272	-
188	H	0.07	A	0.51	-0.04	-	**DhaA103**
191	E	0.1	A	0.55	0.10	-	**DhaA103**
209	L	0.13	I	0.54	0.64	-	-
244	G	0.31	D	0.68	14.59	-	-
271	L	0.09	G	0.57	2.92	-	-
273	Y	0.28	F	0.51	0.30	N41	-

[a] The most conserved residue at a given position of the multiple sequence alignment; [b] Frequency of the most conserved residue at a given position of the multiple sequence alignment
