# Supplementary material for: FireProt: Energy- and Evolution-Based Computational Design of Thermostable Multiple-Point Mutants
Source: PLoS Comput Biol. 2015 Nov 3;11(11):e1004556. doi: 10.1371/journal.pcbi.1004556 (PMC4631455; doi:10.1371/journal.pcbi.1004556)
Supplement: S9 Table — (PDF) [file pcbi.1004556.s012.pdf]

S9 Table. Results of the energy-based analysis of LinA.

Position	Residue	Mutation	FoldX ΔΔG ($kcal.mol^{-1}$)	Rosetta ΔΔG ($kcal.mol^{-1}$)	Interactions	Mutant
3	D	I	-1.234	-3.017		**LinA01**
3	D	L	-1.664	-2.867		
19	D	M	-1.460	-2.388	R79	
127	S	Y	-2.165	-1.952		**LinA01**
145	A	H	-1.254	-4.888		**LinA01**
133	T	I	-2.138	-3.423		**LinA01**
133	T	W	-2.494	-3.308		
133	T	L	-1.323	-1.986		
