# Supplementary material for: FireProt: Energy- and Evolution-Based Computational Design of Thermostable Multiple-Point Mutants
Source: PLoS Comput Biol. 2015 Nov 3;11(11):e1004556. doi: 10.1371/journal.pcbi.1004556 (PMC4631455; doi:10.1371/journal.pcbi.1004556)
Supplement: S10 Table — (PDF) [file pcbi.1004556.s013.pdf]

S10 Table. Results of the consensus analysis of LinA.

Position	Residue	Frequency	[a]Res_TOP	[b]Freq_TOP	FoldX ΔΔG (kcal.mol^{-1})	UniProt database	Mutant
20	K	0.62	Y	0.15	-1.437	Halide-stabilizing	
23	A	0.69	G	0.23	1.778		
32	L	0.62	F	0.38	4.379		
50	Y	0.54	F	0.38	-0.510		**LinA02**
56	A	0.54	I	0.38	5.508		
59	L	0.62	A	0.38	3.642		
68	F	0.62	W	0.31	0.000		**LinA02**
80	L	0.54	V	0.38	1.368		
88	V	0.62	A	0.38	2.694		
96	L	0.77	C	0.15	2.777		
109	I	0.62	V	0.23	0.665		
113	F	0.69	Y	0.23	0.138	Activity decrease	
126	F	0.54	I	0.23	1.624		
131	A	0.62	V	0.15	-1.492		**LinA02**
144	F	0.54	L	0.23	1.316		

[a] The most conserved residue at a given position of the multiple sequence alignment; [b] Frequency of the most conserved residue at a given position of the multiple sequence alignment.
