# Supplementary material for: FireProt: Energy- and Evolution-Based Computational Design of Thermostable Multiple-Point Mutants
Source: PLoS Comput Biol. 2015 Nov 3;11(11):e1004556. doi: 10.1371/journal.pcbi.1004556 (PMC4631455; doi:10.1371/journal.pcbi.1004556)
Supplement: S11 Table — (PDF) [file pcbi.1004556.s014.pdf]

S11 Table. Predicted effects of DhaA115 and LinA01 mutations on its stability.

Origin	Enzyme	Mutation	FoldX ΔΔG (kcal.mol^{-1})	Rosetta ΔΔG (kcal.mol^{-1})	Location	Secondary structure	Flexibility rank [a]	Structural basis of stabilization
Energy-based Approach	DhaA	C128F	-2.2	-8.5	Buried	Sheet	250	Improved packing
		T148L	-2.0	-2.0	Tunnel	Helix	41	Enhanced hydrophobic interactions
		A172I	-2.8	-2.2	Tunnel	Helix	151	Enhanced hydrophobic interactions
		C176F	-2.2	-7.1	Tunnel	Loop	169	Improved packing
		D198W	-1.4	-4.5	Surface	Helix	112	Improved packing
		V219W	-1.8	-3.0	Buried	Helix	109	Improved packing
		C262L	-1.6	-4.9	Buried	Sheet	66	Enhanced hydrophobic interactions
		D266F	-2.3	-2.4	Surface	Sheet	115	Improved packing
	LinA	D3I	-1.2	-3.0	Surface	Helix	151	Improved packing
		S127Y	-2.2	-2.0	Buried	Sheet	58	Improved packing
		T133I	-2.1	-4.9	Buried	Sheet	38	Enhanced hydrophobic interactions
		A145H	-1.3	-3.4	Surface	Loop	91	Improved packing
Evolution-based Approach	DhaA	E20S	0.4	0.5	Surface	Sheet	96	Neutral/electrostatics
		F80R	0.4	1.6	Surface	Helix	98	Neutral/electrostatics
		A155P	-0.8	-0.7	Surface	Loop	4	Increased rigidity

[a] ordered from the most flexible to the most rigid according to average residue B-factors
