# Supplementary material for: FireProt: Energy- and Evolution-Based Computational Design of Thermostable Multiple-Point Mutants
Source: PLoS Comput Biol. 2015 Nov 3;11(11):e1004556. doi: 10.1371/journal.pcbi.1004556 (PMC4631455; doi:10.1371/journal.pcbi.1004556)
Supplement: S12 Table — (PDF) [file pcbi.1004556.s015.pdf]

S12 Table. Examples of methods providing enzymes with outstanding stabilization.

Method	Principle	Experimental work: Protocol	Experimental work: Tested mutants	Experimental work: Number of successful mutants	Enzyme	Stability improvement	Relative activity [a]	Number of mutations	Location of mutations	Reference
Directed evolution										
GSSM	Saturating every position	SSM	121.000	10	Haloalkane dehalogenase	ΔT_m = +18°C	150%	8	Surface and tunnel	[3]
GSSM	Saturating every position	SSM	74.000	10	Xylanese	ΔT_m = +35°C	100%	9	Surface	[4]
RM	Introducing point mutations randomly	epPCR	19.000	16	Phosphite dehydrogenase	ΔT_{50}^{10} = +20°C	160%	12	Buried and surface	[5]
Computational prediction of hotspots										
B-FIT	Targeting the most flexible residues	ISM	19.000	61	Epoxide hydrolase	ΔT_m = +21°C	500%	10	Surface	[6]
HOTSPOT WIZARD	Targeting tunnel residues	SSM	5.000	5	Haloalkane dehalogenase	ΔT_m = +19°C	40%[b]	4	Tunnel	[2]
PISA	Targeting interface residue	ISM	4.000	17	D-tagatose 3-epimerase	ΔT_{50}^{20} = +23°C	64%	8	Dimer interface	[7]
Computational prediction of single-point mutants										
FRESCO	Disulfide bridge design. free energy calculations	SDM	67	24	Epoxide hydrolase	ΔT_m = +36°C	250%	10	Dimer interface and surface	[8]
$ROSETTA_{VIP}$	Improving packing in protein interior by free energy calculation	SDM	6	4	Methionine aminopeptidase	ΔT_m = +18°C	70%	5	Buried	[9]
SCADS	Environmental energy optimization	SDM	1	1	Tobacco 5-epi-aristolochene synthase	ΔT_m = +45°C	2%	12[c]	Buried and surface	[10]
Computational prediction of multiple-point mutants										
FIREPROT	Free energy calculations and consensus design	Gene synthesis	6	4	Haloalkane dehalogenase	ΔT_m = +25°C	128%	11	Surface and buried and tunnel	This study

[a] activity of a mutant compared to the wild-type at temperatures optimal for each protein; [b] activities measured at 37°C in 40% DMSO; [c] predicted single-point mutations were recombined in a multipoint mutant; T_m – melting temperature; T_{50}^X – temperature at which 50% of activity is lost after X minutes of incubation; RM – random mutagenesis; epPCR – error prone polymerase chain reaction; SSM – site saturation mutagenesis; ISM – iterative saturation mutagenesis; SDM – site-directed mutagenesis.
