# Supplementary material for: FireProt: Energy- and Evolution-Based Computational Design of Thermostable Multiple-Point Mutants
Source: PLoS Comput Biol. 2015 Nov 3;11(11):e1004556. doi: 10.1371/journal.pcbi.1004556 (PMC4631455; doi:10.1371/journal.pcbi.1004556)
Supplement: S1 Text — (PDF) [file pcbi.1004556.s016.pdf]

S1 Text. Supporting references

1. Pavlova M, Klvana M, Prokop Z, Chaloupkova R, Banas P, Otyepka M, et al. (2009) Redesigning dehalogenase access tunnels as a strategy for degrading an anthropogenic substrate. Nat Chem Biol. 5: 727-733. doi: 10.1038/nchembio.205. PMID: ISI:000270039900010
2. Koudelakova T, Chaloupkova R, Brezovsky J, Prokop Z, Sebestova E, Hesseler M, et al. (2013) Engineering enzyme stability and resistance to an organic cosolvent by modification of residues in the access tunnel. Angew Chem Int Ed. 52: 1959-1963. doi: 10.1002/anie.201206708. PMID: WOS:000314654000011
3. Gray KA, Richardson TH, Kretz K, Short JM, Bartnek F, Knowles R, et al. (2001) Rapid evolution of reversible denaturation and elevated melting temperature in a microbial haloalkane dehalogenase. Adv Synth Catal. 343: 607-616. doi: 10.1002/1615-4169(200108)343:6/7<607::AID-ADSC607>3.3.CO;2-D.
4. Palackal N, Brennan Y, Callen WN, Dupree P, Frey G, Goubet F, et al. (2004) An evolutionary route to xylanase process fitness. Protein Sci. 13: 494-503. doi: 10.1110/ps.03333504. PMID: WOS:000188411000020
5. Johannes TW, Woodyer RD, Zhao HM. (2005) Directed evolution of a thermostable phosphite dehydrogenase for NAD(P)H regeneration. Appl Environ Microbiol. 71: 5728-5734. doi: 10.1128/aem.71.10.5728-5734.2005. PMID: WOS:000232504000008
6. Gumulya Y, Reetz MT. (2011) Enhancing the thermal robustness of an enzyme by directed evolution: least favorable starting points and inferior mutants can map superior evolutionary pathways. ChemBioChem. 12: 2502-2510. doi: 10.1002/cbic.201100412. PMID: WOS:000297160300018
7. Bosshart A, Panke S, Bechtold M. (2013) Systematic optimization of interface interactions increases the thermostability of a multimeric enzyme. Angew Chem Int Ed. 52: 9673-9676. doi: 10.1002/anie.201304141. PMID: WOS:000323829600015
8. Wijma HJ, Floor RJ, Jekel PA, Baker D, Siewert MJ, Janssen DB. (2014) Computationally designed libraries for rapid enzyme stabilization. Protein Eng Des Sel. 27: 49-58. doi: 10.1093/protein/gzt061. PMID: 24402331
9. Borgo B, Havranek JJ. (2012) Automated selection of stabilizing mutations in designed and natural proteins. Proc Natl Acad Sci U S A. 109: 1494-1499. doi: 10.1073/pnas.1115172109. PMID: WOS:000299731400037
10. Diaz JE, Lin C-S, Kunishiro K, Feld BK, Avrantinis SK, Bronson J, et al. (2011) Computational design and selections for an engineered, thermostable terpene synthase. Protein Sci. 20: 1597-1606. doi: 10.1002/pro.691. PMID: WOS:000294383900013
